# Evaluating the Diagnostic Accuracy of MRI-Derived Prostate-Specific Antigen (PSA) Density in Prostate Cancer Detection and its Association With Tumor Aggressiveness

**DOI:** 10.7759/cureus.74368

**Published:** 2024-11-24

**Authors:** Syed Yousaf Khalid, Tauqir Aslam Waraich, Aiman Elamin

**Affiliations:** 1 Department of Cardiothoracic Surgery, St. James's Hospital, Dublin, IRL; 2 Department of Urology, Letterkenny University Hospital, Letterkenny, IRL; 3 Department of Urology, Sligo University Hospital, Sligo, IRL

**Keywords:** gleason score, mri-based psad, prostate cancer, prostate‑specific antigen, prostate‑specific antigen density

## Abstract

Introduction

Prostate-specific antigen density (PSAD), calculated by dividing serum PSA levels by prostate volume, offers greater specificity and accuracy than serum PSA alone in detecting prostate cancer (PCa). This study aimed to evaluate the diagnostic performance of PSAD in PCa detection across different PSA levels and its correlation with Gleason scores.

Methods

This retrospective, single-center study reviewed data from 154 patients with suspected PCa who underwent prostate MRI between July 2021 and July 2023. Among these, 113 met the inclusion criteria, which required MRI-derived prostate volume measurements, serum PSA levels within three months of biopsy, and transperineal prostate biopsy results. PSAD was calculated by dividing serum PSA levels by prostate volume. Statistical analysis was conducted using STATA/SE 18.0 (StataCorp., College Station, TX, USA). Receiver operating characteristic (ROC) curves identified optimal PSAD cutoff values for PCa detection, and the relationship between PSAD and Gleason scores was analyzed.

Results

Of the 113 patients, 72 (63.72%) were diagnosed with PCa. The overall PSAD cutoff of 0.158 demonstrated a sensitivity of 73.61% and specificity of 92.68%, with an area under the curve (AUC) of 0.83 (95% CI: 0.77-0.90). For patients with PSA levels between 4-10 ng/ml, the optimal PSAD cutoff was 0.155 (sensitivity 65%, specificity 85.19%). For those with PSA levels >10 ng/ml, the cutoff was 0.175 (sensitivity 96.55%, specificity 66.67%). A significant correlation was found between PSAD and Gleason scores (p < 0.001), with higher PSAD values associated with more aggressive cancers.

Conclusion

PSAD demonstrates strong diagnostic accuracy for PCa and is significantly correlated with Gleason scores, suggesting its potential in assessing tumor aggressiveness and guiding clinical decisions.

## Introduction

Prostate cancer (PCa) is among the most commonly diagnosed cancers in men globally, with its incidence rising each year. PCa incidence rates rose by 2-3% per year between 2015 and 2019 [1]. PCa is a significant cause of mortality and morbidity globally, and projections from the Lancet Commission on PCa indicate that the number of new cases is expected to double between 2020 and 2040 [2]. Prostate-specific antigen (PSA) is a widely recognized biomarker in PCa detection, but its specificity is limited, especially within the PSA "gray zone" (typically 4-10 ng/mL), where benign conditions, such as benign prostatic hyperplasia (BPH) and prostatitis, can also elevate PSA level [3]. The resultant ambiguity often leads to overdiagnosis and unnecessary biopsies, emphasizing the need for a more precise diagnostic tool​ [4].

Multiparametric magnetic resonance imaging (mpMRI) of the prostate, utilizing both morphological and functional imaging techniques, has been used for detecting, locating, and staging PCa, as well as for planning patient treatment [5]. PSA density (PSAD), calculated by dividing the PSA level (ng/mL) by prostate volume (mL), has emerged as an improved diagnostic marker, offering greater accuracy and specificity in detecting PCa compared to the PSA level alone [6,7]. Studies have shown that PSAD provides a more reliable distinction between malignant and benign prostate conditions, especially when used in conjunction with imaging techniques such as prostate mpMRI. MRI-derived prostate volumes allow for more precise PSAD calculations, potentially improving the accuracy of PCa detection and reducing the number of unnecessary biopsies​ [5,8].

Studies suggest that PSAD is correlated with tumor aggressiveness, as indicated by the Gleason score, a critical prognostic factor in PCa. This relationship implies that PSAD could aid not only in initial detection but also in evaluating cancer aggressiveness, supporting more personalized treatment planning. However, optimal PSAD cutoff values may vary across populations as studies in different populations have reported slightly different thresholds to optimize the balance of sensitivity and specificity in detecting PCa [7,9,10].

This study aimed to evaluate the diagnostic accuracy of MRI-derived PSAD in detecting PCa across different PSA levels and to explore its correlation with Gleason scores, thus providing further insights into the utility of PSAD as a tool for both PCa detection and assessment of tumor aggressiveness.

## Materials and methods

This retrospective study received approval from the Research Ethics Committee at Letterkenny University Hospital, Letterkenny, Ireland. The Hospital Inpatient Enquiry department provided a list of patients who underwent transperineal prostate biopsies for suspected PCa between July 2021 and July 2023 and then their MRI reports were retrieved and reviewed. Of the 154 cases initially reviewed, 113 met the inclusion criteria and were selected for analysis.

The study included patients who underwent prostate MRI, transperineal prostate biopsy, and serum PSA measurement within three months prior to the biopsy. Exclusion criteria included patients who had received prior treatment for prostate conditions before undergoing MRI, such as surgery, radiation therapy, chemotherapy, or hormonal therapy. Prostate volume measurements were extracted from MRI reports and calculated by the reporting radiologists using the ellipsoid formula:

$Prostate\,volume (mL) = width (cm) &times; length (cm) &times; height (cm) &times; &pi;/6$

Serum PSA levels were extracted from clinical records and the Gleason scores from transperineal prostate biopsy reports. PSAD was calculated by dividing serum PSA levels by the MRI-measured prostate volume.

Categorical variables were reported as frequencies (percentages), while continuous variables were described using the mean (± standard deviation) or median (interquartile range), depending on their distribution. Comparisons of categorical variables were performed using Fisher’s exact test or the chi-square test. For continuous variables, comparisons were made using the independent Student's t-test or the Mann-Whitney U test, as appropriate.

Subgroup analyses of PSAD were performed by dividing patients into three categories based on serum PSA levels: <4 ng/mL, 4-10 ng/mL, and >10 ng/mL. Categorical variables were compared across these subgroups using the chi-square test, while continuous variables were analyzed using one-way ANOVA or the Kruskal-Wallis test, depending on the data distribution. The diagnostic accuracy of PSAD for detecting PCa was assessed using receiver operating characteristic (ROC) curves, with optimal cutoff points determined. The association between PSAD and Gleason scores was analyzed using the Kruskal-Wallis test. Statistical analyses were performed using STATA/SE version 18.0 for Windows (StataCorp., College Station, TX, USA). A p-value of <0.05 was considered statistically significant.

## Results

In this study, 113 patients' data was reviewed, comprising 72 patients (63.72%) diagnosed with PCa and 41 patients (36.28%) without a cancer diagnosis. Patient ages spanned from 43 to 88 years, with an overall mean age of 73.12 ± 8.08 years.

Table 1 summarizes the demographic and clinical characteristics of patients, stratified into cancer (n=72) and non-cancer (n=41) groups. The analysis of serum PSA levels and prostate volume demonstrated significant differences between the cancer and non-cancer groups. Among patients diagnosed with PCa, the median PSA level was 8.85 ng/ml (interquartile range (IQR): 6.73-13.8), and the median prostate volume was 42.5 cm³ (IQR: 31.25-54.75). In contrast, patients without a PCa diagnosis had a median PSA level of 6 ng/ml (IQR: 4.60-7.63) and a higher median prostate volume of 65 cm³ (IQR: 45.5-78.3). PSAD, an important diagnostic indicator, showed a marked difference between the two groups. Patients with PCa had a median PSAD of 0.190 (IQR: 0.14-0.37), which was significantly higher than the median PSAD of 0.094 (IQR: 0.76-1.40) seen in non-cancer patients (p < 0.0001).

**Table 1:** Patient characteristics stratified by cancer and non-cancer groups ^a^: Mann-Whitney U test; IQR: interquartile range; MRI: magnetic resonance imaging; PSA: prostate-specific antigen; PSAD: prostate-specific antigen density; SD: standard deviation

Variables	Cancer (n= 72)	No cancer (n= 41)	Total (n= 113)	Test Statistic	^a^p-value
Age at MRI (years); mean (SD)	75.23 (6.68)	69.43 (9.05)	73.12 (8.08)	z = -3.828	0.0001
Serum PSA levels (ng/ml); median (IQR)	8.85 (6.73-13.8)	6 (4.60-7.63)	7.4 (5.6-11)	z = -4.219	<0.0001
<4	3	8	11	‐	‐
4-10	40	27	67		
>10	29	6	35		
Prostate volume (cm3); median (IQR)	42.5 (31.25-54.75)	65 (45.5-78.3)	48 (34-66)	z = 3.646	0.0003
PSAD; median (IQR)	0.190 (0.14-0.37)	0.094 (0.76-1.40)	0.157 (0.10-0.25)	z = -6.509	<0.0001

The patients were divided into three groups based on their serum PSA levels: less than 4 ng/ml, 4-10 ng/ml, and more than 10 ng/ml. Table 2 presents the demographic and clinical characteristics of patients stratified by serum PSA levels (<4, 4-10, and >10 ng/ml). Of the 72 PCa patients, three had PSA levels below 4 ng/ml, 40 had PSA levels between 4 and 10 ng/ml, and 29 had PSA levels above 10 ng/ml. PSAD showed a marked increase with higher PSA levels, with a median PSAD of 0.0795 (IQR = 0.05-0.11) in the <4 ng/ml group, 0.1313 (IQR = 0.09-0.18) in the 4-10 ng/ml group, and 0.3206 (IQR = 0.19-0.63) in the >10 ng/ml group. Pathology results revealed a higher incidence of PCa in groups with elevated PSA levels, with 83% of patients in the >10 ng/ml group diagnosed with cancer, compared to only 27% in the <4 ng/ml group.

**Table 2:** Patient characteristics stratified by PSA levels (<4, 4–10, and >10 ng/mL) ^a^: Kruskal-Wallis test; IQR: interquartile range; MRI: magnetic resonance imaging; PSA: prostate-specific antigen; PSAD: prostate-specific antigen density; SD: standard deviation

Variables	<4 ng/ml	4-10 ng/ml	>10 ng/ml	Test statistics	^a^p-value
Age at MRI (years), mean (SD)	64.89 (11.12)	72.30 (7.69)	77.30 (4.77)	χ² = 20.462	0.0001
Prostate volume (cm3) Median (IQR)	33.00 (27-44)	51.00 (37-65)	50.00 (33.2-75)	χ² = 5.703	0.0577
PSAD Median (IQR)	0.0795 (0.05-0.11)	0.1313 (0.09-0.18)	0.3206 (0.19-0.63)	χ² = 50.838	0.0001
Pathology, n	11	67	35	‐	
Cancer	3	40	29		
No cancer	8	27	6		
Gleason score, n					
3+3	2	19	9		
3+4	0	14	4		
3+5	1	1	2		
4+3	0	4	4		
4+4	0	1	3		
4+5	0	1	3		
5+4	0	0	2		
5+5	0	0	2		

**Figure 1:**
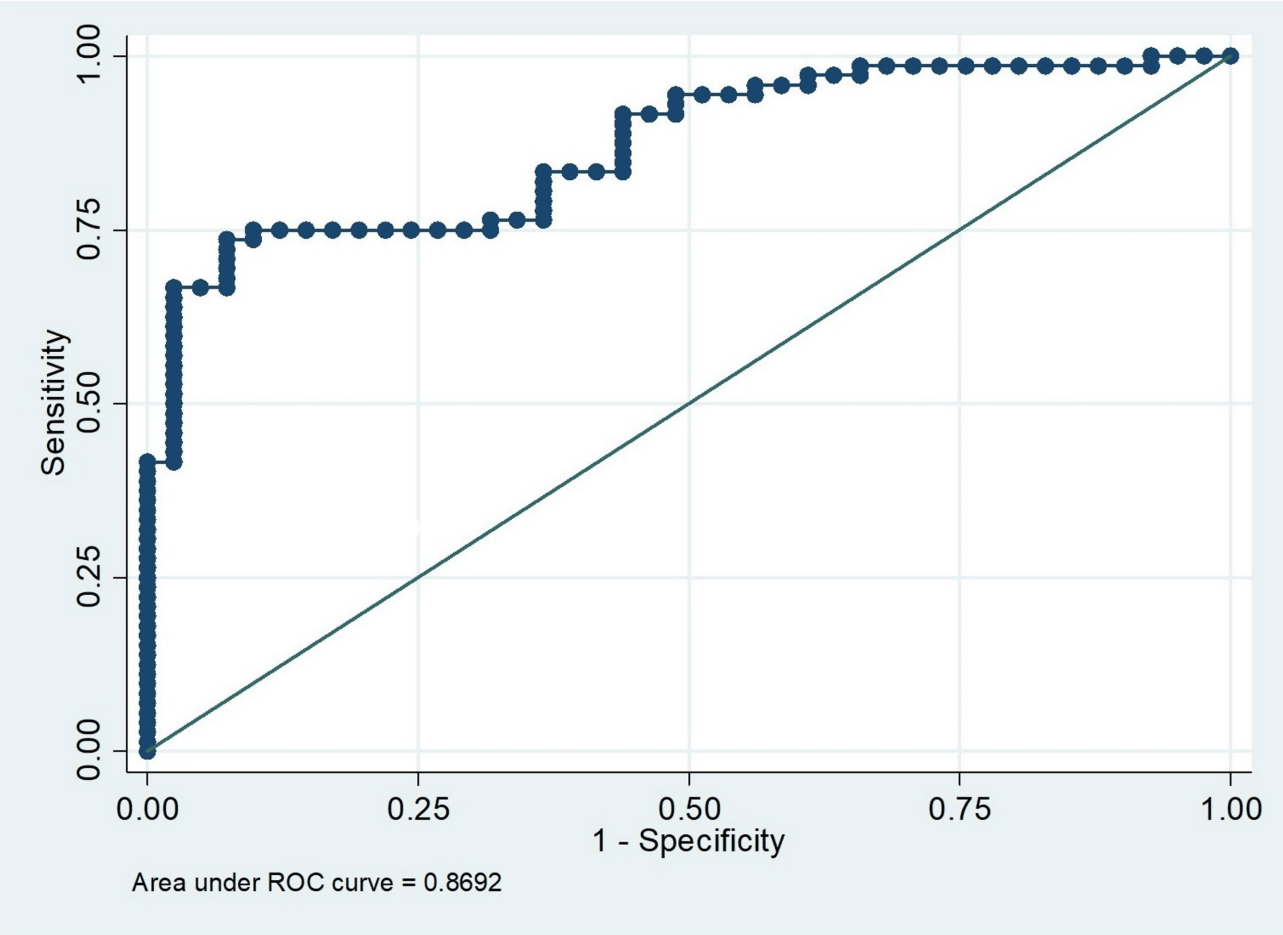
Receiver operating characteristic curves for PSAD in differentiating PCa ROC: receiver operating characteristic; PSAD: prostate-specific antigen density; PCa: prostate cancer

The overall PSAD cutoff value was 0.158, achieving a sensitivity of 73.61% and a specificity of 92.68%, with an AUC of 0.83 (95% CI: 0.77-0.90), as detailed in Table 3. In subgroup analyses, the optimal cutoff for patients in the 4-10 ng/ml range was 0.155, resulting in a sensitivity of 65% and a specificity of 85.19%, with an AUC of 0.81. For patients with levels >10 ng/ml, the ideal cutoff was 0.175, with a sensitivity of 96.55%, a specificity of 66.67%, and an AUC of 0.90. Figure 1 displays the ROC curves for PSAD in the identification of PCa.

**Table 3:** PSAD cutoff threshold for PCa detection in all patients, those with PSA levels ranging from 4-10 ng/ml and greater than 10 ng/ml PCa: prostate cancer; CI: confidence interval; PSA: prostate-specific antigen; PSAD: prostate-specific antigen density; PPV: positive predictive value; NPV: negative predictive value

PSA level (ng/ml)	Cutoff	Sensitivity (%) (95% CI)	Specificity (%) (95% CI)	PPV (%) (95%CI)	NPV (%) (95%CI)	Accuracy (%) (95%CI)
All	0.158	73.61 (62.42-82.41)	92.68 (80.57-97.48)	94.64 (85.39-98.16)	66.67 (53.72-77.51)	80.53 (72.28-86.78)
4-10	0.155	65.00 (50.22-79.78)	85.19, (71.79 - 98.59)	86.67 (74.50 - 98.83)	62.16 (46.53 - 77.79)	73.13 (62.52 - 83.75)
>10	0.175	96.55 (82.24-99.91)	66.67 (22.28 – 95.67)	93.33 (77.93-99.18)	80.00 (28.36-99.49)	91.43 (76.94-98.20)

**Figure 2:**
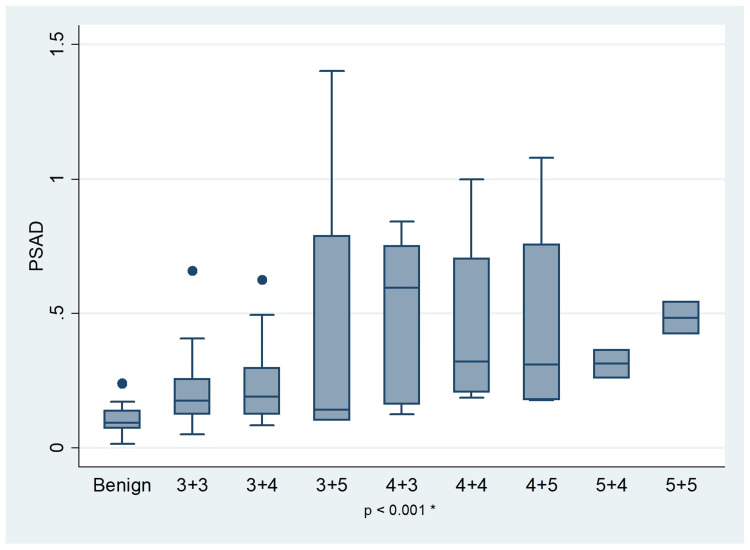
Correlation between PSAD and Gleason score PSAD: prostate-specific antigen density; *:Kruskal-Wallis test (χ² = 51.591)

Figure 2 presents box plots of PSAD values categorized by Gleason scores. The median PSAD values for patients with various Gleason scores were as follows: 0.18 for 3+3, 0.19 for 3+4, 0.14 for 3+5, 0.60 for 4+3, 0.32 for 4+4, 0.31 for 4+5, 0.31 for 5+4, and 0.48 for 5+5. These results highlight a trend of increasing PSAD with higher Gleason scores, particularly in more aggressive tumor categories. A statistically significant correlation between PSAD and Gleason scores was observed (p < 0.001).

## Discussion

The findings of this study emphasize the diagnostic value of PSAD in the detection and assessment of PCa. While serum PSA levels remain a widely utilized biomarker for PCa screening, their lack of specificity, particularly in the gray zone (4-10 ng/mL), limits their effectiveness and often results in unnecessary biopsies. In our cohort, median serum PSA levels were significantly higher (p-value <0.0001) in PCa patients (8.85 ng/mL) compared to non-cancer patients (6 ng/mL). Furthermore, the median prostate volume in patients with PCa was significantly lower at 42.5 cm³ compared to 65 cm³ in patients without cancer. These findings align with prior research indicating that BPH can elevate serum PSA levels [3,11].

The introduction of PSAD, calculated as serum PSA divided by prostate volume, represents a critical advancement in improving the specificity of PSA screening. Initially proposed by Benson et al. [12,13], PSAD has demonstrated greater accuracy than PSA levels alone for the detection of PCa in numerous studies [6,7,11]. While most research has utilized transrectal ultrasound (TRUS) for prostate volume measurement, our study employed MRI, which may offer a more precise assessment. Previous studies have also demonstrated that prostate volume measurement by MRI is superior to TRUS in accuracy [14-16]. Previous studies have suggested that the PSAD cut-off typically ranges from 0.15 to 0.2, which aligns with our study’s ideal cut-off of 0.155, falling within this established range.

Previous studies have shown that PSA levels vary between different populations, leading to differences in PSAD values and optimal cutoff points as well. Saema et al. [17] identified an optimal PSAD cut-off of 0.15 in the Thai population, yielding a sensitivity of 78% and specificity of 43% for patients with PSA levels between 4 and 10 ng/mL. In contrast, our study found a PSAD cut-off of 0.155, yielding a sensitivity of 65% but a notably higher specificity of 85.19%. This difference may be attributed to variations in population characteristics or diagnostic methods.

In this study, the optimal PSAD cutoff point for distinguishing PCa was 0.158, with a sensitivity of 73.61%, specificity of 92.68%, positive predictive value (PPV) of 94.64%, and negative predictive value (NPV) of 66.67%. The AUC was 0.83 (95% CI: 0.77-0.90). In a similar study by Aphinives et al. [9], the optimal cutoff point of PSAD for discrimination of PCa was 0.16 (81.40% sensitivity, 54.70% specificity, 52.70% PPV, 82.50% NPV), and the AUC was 0.680 (95%CI: 0.609-0.751). Variations in ethnicity may account for the differences in the results compared to previous studies, that reported PSAD cut-off of 0.11, 0.11, 0.16, and 0.70 in Brazilian, Iranian, Thai, and Indonesian patients, respectively [7,9,18,19].

In our subgroup analysis of patients with serum PSA levels categorized as <4 ng/mL, 4-10 ng/mL, and >10 ng/mL, only three patients with PSA levels below 4 ng/mL were diagnosed with PCa. This observation is consistent with findings from Thompson et al. [20], who reported that 10-27% of men aged 62-91 years with PSA levels of 4.0 ng/mL or lower were diagnosed with PCa. Our study established optimal PSAD cutoff values for detecting PCa among patients with PSA levels of 4-10 ng/mL and >10 ng/mL, identified as 0.155 (65% sensitivity and 85.19% specificity) and 0.175 (96.55% sensitivity and 66.67% specificity), respectively. These findings are consistent with prior research, particularly concerning patients with PSA levels between 4-10 ng/mL. Lin et al. [10] identified a PSAD cutoff of 0.15 for PSA levels between 2.5-10 ng/mL and 0.33 for PSA levels between 10-20 ng/mL. Aphinives et al. [9] similarly reported cut-offs of 0.16 for PSA levels of 4-10 ng/mL and 0.33 for levels >10 ng/mL. 

Notably, our study revealed a significant association between PSAD and Gleason scores in PCa patients (p < 0.001), reinforcing findings from Karademir et al. [21] and Aphinives et al [9]. This association suggests that PSAD may serve as a prognostic tool, aiding in the assessment of tumor aggressiveness and guiding treatment decisions.

Limitations

Our study had several limitations. First, its retrospective design may have introduced inherent biases, potentially limiting the generalizability of the findings. Second, as a single-center study, the results may not be fully representative of broader populations, especially given that the majority of participants were over 70 years old. This may limit the applicability of results to younger cohorts or those with differing risk profiles. Third, the relatively small sample size reduced the statistical power, which could affect the robustness of the conclusions. To address these issues, future studies should explore multicenter and prospective designs to validate these findings and investigate the impact of demographic and methodological variations on PSAD’s diagnostic accuracy.

## Conclusions

This study highlights the value of MRI-derived PSAD as a promising tool for improving PCa diagnosis and evaluating tumor aggressiveness. PSAD demonstrates greater accuracy in distinguishing cancerous from non-cancerous cases compared to serum PSA alone, particularly within intermediate PSA ranges. By using optimized PSAD cutoff values, clinical decisions can be better informed, helping to minimize unnecessary procedures and ensure timely intervention. Additionally, the strong association observed between elevated PSAD values and more aggressive cancer emphasizes PSAD’s potential for assessing tumor aggressiveness. These findings support the integration of PSAD in clinical settings to enhance diagnostic precision and enable more personalized treatment approaches for patients with suspected PCa.
